# Education in early life markedly reduces the probability of cognitive impairment in later life in Colombia

**DOI:** 10.1038/s41598-020-74822-2

**Published:** 2020-10-19

**Authors:** Gary O’Donovan, Mark Hamer, Olga L. Sarmiento, Philipp Hessel

**Affiliations:** 1 Facultad de Medicina, Universidad de Los Andes, Carrera 1, 18A-12, 111711 Bogotá, Colombia; 2 Institute of Sport, Exercise and Health, University College London, London, UK; 3 Escuela de Gobierno Alberto Lleras Camargo, Universidad de Los Andes, Bogotá, Colombia

**Keywords:** Dementia, Epidemiology

## Abstract

The objective of this study was to investigate associations between education in early life and cognitive impairment in later life in Colombia. Participants were community-dwelling adults aged 60 years or older from the National Study of Health, Wellbeing and Ageing. Trained interviewers administered a shorter version of the mini-mental state examination. Cognitive impairment was defined as the lowest tertile in the main analysis and as a score of 12 or less out of 19 in the sensitivity analysis. Logistic regression models were adjusted for education, other early life characteristics, and later life characteristics. The prevalence of cognitive impairment was 17.93% in the main analysis (n = 16,505). Compared with participants with no education, the fully adjusted odds ratio for cognitive impairment was 0.57 (95% confidence interval: 0.52, 0.63) in those with some primary education and 0.29 (95% confidence interval: 0.25, 0.34) in those with some secondary education or more. The population attributable fraction for education suggests that at least 10% of cases of cognitive impairment would be eliminated if all children received an education. Similar results were observed in the sensitivity analysis (n = 20,174). This study suggests that education in early life markedly reduces the probability of cognitive impairment in late life in Colombia.

## Introduction

The global burden of disease attributable to mental disorders is rising to such an extent that poor mental health is regarded as a threat to sustainable development^1^. Mental health is a fundamental human right for all people, yet the burden of mental and substance use disorders is high in young people and the burden of Alzheimer’s disease and other dementias is high in older people^1,2^. In the Lancet Commission on global mental health and sustainable development, it is proposed that mental health in adults be understood from a life course perspective, with education in early life playing an important role in building cognitive reserve and reducing the risk of dementia in later life^1^. It is plausible that education reduces the risk of mild cognitive impairment and dementia^3,4^; however, more research is required to understand the association between education in early life and mild cognitive impairment in later life in Colombia and elsewhere in Latin America^5,6^. Inverse associations between education and cognitive impairment have been observed in older adults in Bogotá and five other cities in Colombia^7–9^; however, these analyses were not adjusted for early life characteristics or later life characteristics that may be associated with mental health such as nutrition, physical activity and socioeconomic status^1,6,10–12^. The studies in Bogotá and elsewhere in Colombia included between 1235 and 2000 participants^7–9^ and were too small to estimate the population attributable fraction for education, which is the proportion of cases of cognitive impairment that would be eliminated if all children received an education^13^. The National Survey of Health, Wellbeing and Ageing in Colombia (SABE Colombia, according to its initials in Spanish) is a nationwide study of adults older than 60 years of age^14^. The main objective of the present study is to investigate whether education in early life reduces the probability of cognitive impairment in participants in SABE independent of a range of early life and later life characteristics that may be associated with mental health. The secondary objective is to estimate the proportion of cases of cognitive impairment that are attributable to education.

## Methods

This study was conducted in accordance with the STROBE Statement and includes the items that should be included in reports of observational studies^15^.

### Participants

The National Survey of Health, Wellbeing and Ageing in Colombia is described in detail elsewhere^14^. Briefly, the target population was all adults aged 60 years or older living in households. Participants were selected using a multistage area probability sampling design and there were four selection stages: municipalities, blocks, housing units, and households. The response rate was around 62% in urban areas, around 77% in rural areas, and around 70% overall^14^. Data were collected across all departments (that is, states) and the final sample was deemed to be representative of the population of older adults living in households in Colombia^14^. Standard operating procedures were written for all procedures in the protocol and a study manual was written to guide fieldwork. Research and field staff were trained in all aspects of data collection and the core research staff trained the interviewers using role-playing^14^. The trained interviewers conducted face-to-face interviews in the participant’s home between April and September 2015^14^. Volunteers completed the shorter version of the Folstein Mini-Mental State Examination (MMSE) described below and were invited to participate in the interview if they had a score of 13 or more^14^. Otherwise, a friend or family member was invited to complete the interview on behalf of the participant. Such a proxy obtained a score of 13 or more and completed the interview in 17.5% of cases^14^. Institutional review boards of *Universidad de Caldas* and *Universidad del Valle* approved the study and all participants gave written informed consent.

### Dependent variable

The dependent variable was cognitive impairment. The versions of the MMSE used in Latin America and the Caribbean are shorter than the original version in an attempt to reduce the low literacy bias^16^. The shorter version of the MMSE used in SABE Colombia has six questions and participants were asked: to state the date and the day of week (4 points); to repeat and remember three words (3 points); to state in reverse order the numbers 1, 3, 5, 7, 9 (5 points); to take a piece of paper in their right hand, fold it in half using both hands, and put it on their lap (3 points); to reiterate the three words given earlier (3 points); and, to copy a drawing of two overlapping circles (1 point). Many English versions and several Spanish versions of the MMSE have been used to screen for mild cognitive impairment in older adults^17^, with sensitivity of 45% (95% confidence interval (CI): 39, 52) and specificity of 80% (95% CI: 75, 84) in the good quality studies^17,18^. Sensitivity and specificity may be higher when lower thresholds are used to screen for mild cognitive impairment in Spanish speaking populations with lower levels of education^19,20^. A score of 12 or less out of 19 was used to screen for mild cognitive impairment in SABE Colombia^14^. The shorter version of the MMSE used in SABE Colombia has been validated in a study of 1,301 adults aged 60 years or older living in households in Chile^21^. The prevalence of mild cognitive impairment was 10.7% using the threshold of 12 or less out of 19 in the shorter version of the MMSE and 8.1% using the threshold of 6 or more out of 33 in the criterion measure^21^, which was the Short Portable Mental Status Questionnaire^22^.

### Independent variables

The trained interviewers followed a questionnaire and asked about age, sex, education, the economic situation of the family during childhood, self-rated health during childhood, current income, civil status, cigarette smoking, alcohol drinking, self-rated nutritional status, and physical activity. Participants were asked about the highest level of education they had achieved, and three groups were created: no education; some primary education; and, some secondary education or more. Interviewees were asked to state whether the economic situation of their family during their first 15 years of life was bad, normal or good. Interviewees were asked to state whether their health during their first 15 years of life was bad, normal or good. Participants were asked about their current individual income according to multiples of the minimum wage. Participants were asked about their current civil status, and two groups were created: not married or with partner; and, married or with partner. Participants were asked about cigarette smoking, and two groups were created: never smoker; and, current or ex-smoker. Participants were asked about alcohol drinking in the last month, and two groups were created: non-drinker; and, drinker. Interviewees were asked to rate their current state of nutrition as serious malnutrition, moderate malnutrition, or good nutrition. Participants were categorized as physically active if they reported taking part in sport or exercise at least three times per week or if they reported walking between 9 and 20 blocks (1.6 km) at least three times per week. The trained interviewers also measured height. The proxy was not asked about the economic situation of the family during childhood, self-rated health during childhood, or self-rated nutritional status.

### Statistical analysis

All analyses were performed using Stata MP version 15.1 for Mac (StataCorp, Texas, USA). Logistic regression was used to investigate associations between education and cognitive impairment. In other studies, many thresholds have been used to screen for mild cognitive impairment^17^. In the present study, the threshold for mild cognitive impairment was the lowest tertile of the shorter version of the MMSE in the main analysis and a score of 12 or less in the sensitivity analysis. Logistic regression models were adjusted for age, sex, height quintile, education, economic situation of the family during childhood, self-rated health during childhood, current individual income, civil status, cigarette smoking, alcohol drinking, self-related nutritional status, and physical activity. Age was modelled as a continuous variable. All other covariates were modelled as categorical variables. The *margins* and *atmeans* commands in Stata were used to investigate the probability of cognitive impairment according to education level, with the covariates fixed at their means. The *punaf* command in Stata was used to estimate the population attributable fraction for education, adjusting for the covariates^13^.

## Results

There were 23,694 participants in SABE; 16,505 were included in the main analysis and 20,174 were included in the sensitivity analysis in the present study. Table S1 in the online supplement shows selected participants’ characteristics in SABE and in the sub-samples. For example, the score on the shorter version of the MMSE was 14.9 ± 3.9 in SABE (mean ± SD), 16.6 ± 1.9 in the main analysis (p < 0.001 vs. SABE), and 15.4 ± 3.5 in the sensitivity analysis (p < 0.001 vs. SABE); Age was 70.8 ± 8.2 years in SABE, 68.9 ± 6.9 years in the main analysis (p < 0.0.01 vs. SABE), and 70.0 ± 7.7 years in the sensitivity analysis (p < 0.001 vs. SABE); And, the proportion of males was 42.68% in SABE, 45.27% in the main analysis (p < 0.001 vs. SABE), and 44.18% in the sensitivity analysis (p < 0.001 vs. SABE). The proportion of participants who reported some primary education was 57.04% in SABE, 59.02% in the main analysis (p < 0.001 vs. SABE), and 57.47% in the sensitivity analysis (p = 0.22 vs. SABE). The number of years of education was 3.59 ± 3.82 in SABE, 4.07 ± 4.00 in the main analysis (p < 0.001 vs. SABE), and 3.68 ± 3.85 in the sensitivity analysis (p < 0.05 vs. SABE).

Table 1 shows the score on the shorter version of the MMSE, age, proportion of males, level of education and all other variables in the sub-samples in the present study. The economic situation of the family during childhood was “bad” in 16.85% of participants, self-rated health during childhood was “good” in 89.7% of participants, and nutritional status was “good” in 72.28% of participants in the main analysis. These variables were not included in the sensitivity analysis because the proxy interviewee was not asked such questions. Current income was less than minimum wage in 55.64% of participants in the main analysis and 57.60% of participants in the sensitivity analysis. The proportion of participants married or with partner was 57.60% in the main analysis and 54.93% in the sensitivity analysis. The proportion of current or ex-smokers was 52.61% in the main analysis and 52.60% in the sensitivity analysis. Some 14% of participants in the main analysis and 12.74% of participants in the sensitivity analysis reported drinking alcohol. More than 50% of the participants in each of the sub-samples reported being physically active. Table S2 and Table S3 in the online supplement show participants’ characteristics in the sub-samples according to education levels. Compared with participants with no education, the score on the shorter version of the MMSE was higher and age was lower in those with some primary or some secondary education in both the main analysis and the sensitivity analysis. There were favourable differences in early life characteristics according to education level in the main analysis. And, there were favourable differences in later life characteristics according to education level in both the main analysis and the sensitivity analysis. For example, the proportion of physically active individuals in the main analysis was 55.30% in those with no education, 57.75% in those with some primary education (p < 0.001 vs. those with no education), and 66.88% in those with some secondary education or more (p < 0.001 vs. those with no education and those with some primary education).

**Table 1:** Participants’ characteristics.

Variable	Main analysis (n = 16,505)	Sensitivity analysis (n = 20,174)
Shorter MMSE score (mean ± SD)	16.6 ± 1.9	15.4 ± 3.5
Age, years (mean ± SD)	68.9 ± 6.9	70.0 ± 7.7
Male, %	45.27	44.18
**Height, cm (mean ± SD)**		
Quintile 1	144.92 ± 2.61	144.76 ± 2.7
Quintile 2	151.08 ± 1.40	151.04 ± 1.40
Quintile 3	155.94 ± 1.41	155.95 ± 1.41
Quintile 4	161.96 ± 2.0	161.93 ± 2.0
Quintile 5	170.23 ± 3.75	170.26 ± 3.78
**Education, %**		
None	15.79	20.91
Some primary	59.02	57.47
Some secondary or more	25.19	21.63
**Economic situation of family during childhood, %**		
Bad	16.85	-
Normal	42.42	-
Good	40.73	-
**Self-rated health during childhood, %**		
Bad	1.45	-
Normal	8.85	-
Good	89.7	-
**Current income, %**		
None	14.16	15.33
Less than minimum wage	55.64	57.60
Minimum wage	15.07	13.82
More than 1 to 2 times minimum wage	9.50	8.44
More than 2 to 3 times minimum wage	2.99	2.57
More than 3 to 4 times minimum wage	1.45	1.23
More than 4 times minimum wage	1.19	1.00
**Civil status, %**		
Not married or with partner	42.40	45.07
Married or with partner	57.60	54.93
**Cigarette smoking, %**		
Never smoker	47.39	47.40
Current or ex-smoker	52.61	52.60
**Alcohol drinking, %**		
Non-drinker	85.82	87.26
Drinker	14.18	12.74
**Self-rated nutritional status, %**		
Serious malnutrition	1.99	-
Moderate malnutrition	25.73	-
Good nutrition	72.28	-
**Physically active, %**		
No	40.34	44.36
Yes	59.66	55.64

Cognitive impairment was defined as the lowest tertile of the shorter version of the mini-mental state examination (MMSE) in the main analysis and as a score of 12 or less out of 19 in the sensitivity analysis. Economic situation of family during childhood, self-rated health during childhood, and self-rated nutritional status could not be included in the sensitivity analysis because the proxy interviewee was not asked about these variables.

The prevalence of cognitive impairment was 17.93% in the main analysis, where the threshold for mild cognitive impairment was the lowest tertile of the shorter version of the MMSE. Table 2 shows the probability of cognitive impairment in the 16,505 older adults in the main analysis. The fully adjusted odds ratio (95% confidence interval) was 1.03 (1.03, 1.04) for age and 1.06 (0.93, 1.20) for female sex. Compared with participants with no education, the fully adjusted odds ratio for cognitive impairment was 0.57 (0.52, 0.63) in those with some primary education and 0.29 (0.25, 0.34) in those with some secondary education or more. Greater income, good nutrition and physical activity were also associated with lower probability of cognitive impairment. The prevalence of cognitive impairment was 16.16% in the sensitivity analysis, where the threshold for mild cognitive impairment was a score of 12 or less on the shorter version of the MMSE. Table 2 also shows the probability of cognitive impairment in the 20,174 older adults in the sensitivity analysis. The fully adjusted odds ratio was 1.09 (1.09, 1.10) for age and 0.82 (0.72, 0.93) for female sex. Compared with participants with no education, the fully adjusted odds ratio for cognitive impairment was 0.33 (0.30, 0.36) in those with some primary education and 0.12 (0.10, 0.14) in those with some secondary education or more. Greater income, being married or with partner, being a smoker or ex-smoker, drinking alcohol, and being physically active were also associated with lower probability of cognitive impairment.

**Table 2:** Odds of cognitive impairment.

Independent variable	Main analysis (n = 16,505)	Sensitivity analysis (n = 20,174)
Age, years	1.03 (1.03, 1.04)	1.09 (1.09, 1.10)
Female sex	1.06 (0.93, 1.20)	0.82 (0.72, 0.93)
**Height**		
Quintile 1	Reference	Reference
Quintile 2	0.95 (0.84, 1.09)	0.81 (0.72, 0.92)
Quintile 3	0.95 (0.83, 1.08)	0.83 (0.73, 0.95)
Quintile 4	0.92 (0.79, 1.06)	0.78 (0.67, 0.91)
Quintile 5	0.93 (0.78, 1.10)	0.75 (0.63, 0.90)
**Education**		
None	Reference	Reference
Some primary	0.57 (0.52, 0.63)	0.33 (0.30, 0.36)
Some secondary or more	0.29 (0.25, 0.34)	0.12 (0.10, 0.14)
**Economic situation of family during childhood**		
Bad	Reference	-
Normal	1.05 (0.93, 1.19)	-
Good	1.25 (1.11, 1.41)	-
**Self-rated health during childhood**		
Bad	Reference	-
Normal	0.89 (0.63, 1.26)	-
Good	0.80 (0.58, 1.10)	-
**Current income**		
None	Reference	Reference
Less than minimum wage	1.04 (0.92, 1.17)	0.70 (0.63, 0.78)
Minimum wage	0.95 (0.82, 1.11)	0.46 (0.39, 0.55)
More than 1 to 2 times minimum wage	0.81 (0.67, 0.98)	0.44 (0.35, 0.55)
More than 2 to 3 times minimum wage	0.77 (0.56, 1.07)	0.34 (0.21, 0.54)
More than 3 to 4 times minimum wage	0.46 (0.26, 0.81)	0.27 (0.12, 0.64)
More than 4 times minimum wage	0.22 (0.09, 0.55)	0.32 (0.13, 0.82)
**Civil status**		
Not married or with partner	Reference	Reference
Married or with partner	0.98 (0.89, 1.07)	0.74 (0.68, 0.81)
**Cigarette smoking**		
Never smoker	Reference	Reference
Current or ex-smoker	1.07 (0.98, 1.17)	0.89 (0.81, 0.97)
**Alcohol drinking**		
Non-drinker	Reference	Reference
Drinker	0.94 (0.83, 1.08)	0.68 (0.58, 0.81)
**Self-rated nutritional status**		
Serious malnutrition	Reference	-
Moderate malnutrition	0.79 (0.61, 1.02)	-
Good nutrition	0.75 (0.58, 0.97)	-
**Physically active**		
No	Reference	Reference
Yes	0.90 (0.83, 0.98)	0.60 (0.55, 0.65)

Values are mutually adjusted odds ratio (95% confidence interval). Cognitive impairment was defined as the lowest tertile of the shorter version of the mini-mental state examination (MMSE) in the main analysis and as a score of 12 or less out of 19 in the sensitivity analysis. Economic situation of family during childhood, self-rated health during childhood, and self-rated nutritional status could not be included in the sensitivity analysis because the proxy interviewee was not asked about these variables.

**Figure 1:**
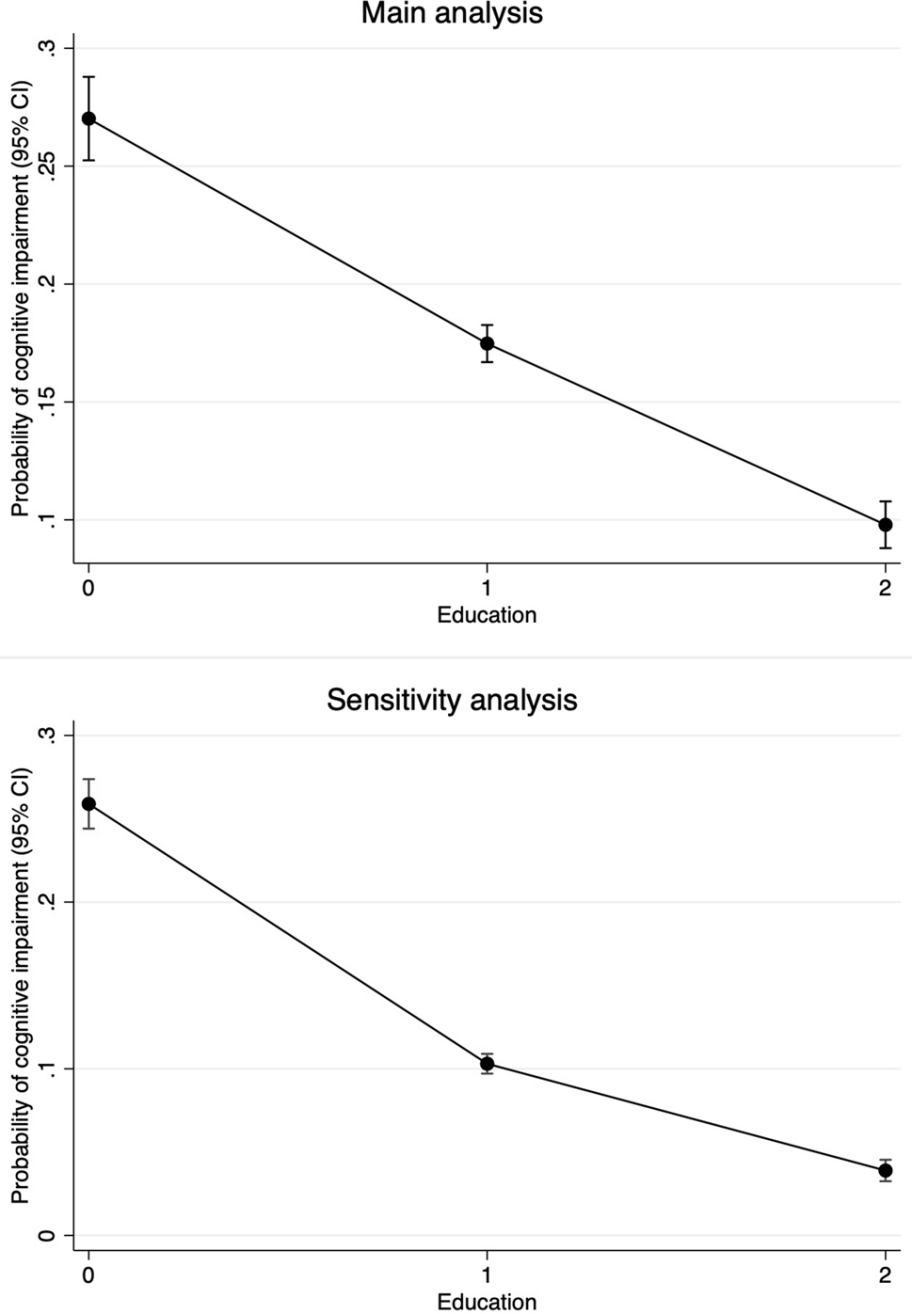
The probability of cognitive impairment according to education level, with the covariates fixed at their means. 0 is no education. 1 is some primary education. 2 is some secondary education or more. Sample size was 16,505 in the main analysis and cognitive impairment was defined as the lowest tertile of the shorter version of the mini-mental state examination. Sample size was 20,174 in the sensitivity analysis and cognitive impairment was defined as a score of 12 or less out of 19.

Figure 1 shows the probability of cognitive impairment according to education level, with the covariates fixed at their means. In the main analysis, the probability of cognitive impairment was 0.27 (0.25, 0.29) in those with no education, 0.17 (0.17, 0.18) in those with some primary education, and 0.10 (0.09, 0.11) in those with some secondary education or more. In the sensitivity analysis, the probability of cognitive impairment was 0.26 (0.24, 0.27) in those with no education, 0.10 (0.10, 0.11) in those with some primary education, and 0.04 (0.03, 0.05) in those with some secondary education or more.

In the main analysis, the population attributable fraction for education was estimated while adjusting for age, sex, height, education, economic situation of the family during childhood, self-rated health during childhood, current individual income, civil status, cigarette smoking, alcohol drinking, self-related nutritional status, and physical activity (n = 16,505). Theoretically, 10% (9, 12) of cases of cognitive impairment would be eliminated if all children received an education (i.e., some primary education or some secondary education or more). Some 43% (37, 48) of cases would be eliminated if all children received an education that was secondary education or more. In the sensitivity analysis, the population attributable fraction was estimated while adjusting for age, sex, height, education, current individual income, civil status, cigarette smoking, alcohol drinking, and physical activity (n = 20,174). Theoretically, 25% (23, 27) of cases would be eliminated if all children received an education and 65% (59, 70) of cases would be eliminated if that education was secondary education or more.

## Discussion

The main objective of this study was to investigate whether education in early life reduced the probability of cognitive impairment in older adults in Colombia. We found that, compared with participants with no education, the probability of cognitive impairment was lower in those with some primary education and lower still in those with some secondary education or more. Any education markedly reduced the probability of cognitive impairment, whether cognitive impairment was defined as the lowest tertile of the mental state examination or as a score of 12 or less. Magnetic resonance imaging studies suggest that it is plausible that education reduces the risk of dementia because greater education in early life is associated with greater grey matter volume in middle age^3^ and greater lifespan mental activity is associated with lower hippocampal atrophy in older age^4^. The secondary objective of this study was to estimate the proportion of cases of cognitive impairment that were attributable to education. We found that at least 10% of cases of cognitive impairment would be eliminated if all children received an education.

To the best of our knowledge, this is the largest and most robust study of education and cognitive impairment in older adults in Colombia^7–9^. Inverse associations between education and cognitive impairment have been reported in 1,611 older adults in five cities of Colombia^7^, in 1,235 older adults in the city of Bogotá in Colombia^8^, and in 2,000 older adults in the city of Bogotá in Colombia^9^; However, these analyses were only adjusted for age and sex^7,9^, and for age, sex and “comorbidities”^8^. The present study is also one of the largest studies of its kind in Latin America^6,23–27^. The 10/66 Dementia Research Group includes data from “representative populations” from Cuba, Dominican Republic, Mexico, Peru, Puerto Rico and Venezuela (the Latin American sample) and from China and India^28^. The data from the 12,865 older adults in the Latin American sample suggest that the prevalence of low education is around 70% and the population attributable fraction for dementia is around 11%^6^. In the present study, the prevalence of low education in Colombia was around 75–80% and the population attributable fraction for cognitive impairment was around 10–25%. These studies suggest that there should be more emphasis on education in early life in subsequent iterations of the mental health act of Colombia as part of a policy to improve mental health across the life course^29,30^. While it is plausible that education reduces the risk of dementia^3,4^, it is also possible that people with more education in early life adopt lifestyles that are conducive to brain health in later life^1^. Indeed, compared with participants in the present study with no education, the proportion of smokers was lower in those with some primary education and lower still in those with some secondary education or more. And, compared with participants with no education, the proportion of physically active individuals was higher in those with some primary education and higher still in those with some secondary education or more.

The present analysis was adjusted for a range of covariates that may be associated with mental health^1,6,10–12^. The observed associations were in the expected directions, except cigarette smoking^6^. More research is needed to understand the association between alcohol and dementia^31^. There is growing evidence that cardiovascular disease risk factors deteriorate many years before the onset of dementia and that cardiovascular disease risk factors are not associated with cognitive decline in older adults^32–34^. In the present sample, 2088 participants reported a diagnosis of heart disease and the fully adjusted odds ratio for cognitive impairment was 0.97 (0.86, 1.10). In the present sample, body mass index was measured in 20,124 participants, and body mass index was not associated with cognitive impairment in the fully adjusted model (data not shown). In the present sample, blood samples were obtained from 3530 participants after an overnight fast, and total cholesterol concentration, HDL-cholesterol concentration, and LDL-cholesterol concentration were not associated with cognitive impairment in the fully adjusted model (data not shown).

This study has some limitations. Longitudinal studies are needed to clarify the associations between early life characteristics, later life characteristics, and cognitive impairment; however, it is reasonable to assume that education preceded cognitive impairment even in this cross-sectional study. The thresholds used in screening tests of cognitive impairment are often arbitrary^17^; however, we used a sensitivity analysis to demonstrate the robustness of the assessment. The shorter version of the MMSE used in SABE Colombia is a valid screening tool^21^, but it is not a clinical diagnosis of cognitive impairment. Some variables were self-reported and are subject to biases. There was no measure of the genetic factors that may play a role in determining individual risk of dementia^35^. There was no measure of quality of education in the present study; however, it is encouraging that any amount of education markedly reduced the probability of cognitive impairment.

## Conclusion

Little was known about the association between education in early life and cognitive impairment in later life in Colombia because there were no nationwide surveys, only small studies in different cities. The National Survey of Health, Wellbeing and Ageing in Colombia is a representative study of 23,694 older adults. To the best of our knowledge, the present study is the largest report of an inverse association between education and cognitive impairment in older adults in Colombia and elsewhere in Latin America & the Caribbean. There should be more emphasis on education in early life in subsequent iterations of the mental health act of Colombia as part of a policy to improve mental health across the life course.

## Supplementary information

Supplementary information
