# Supplementary material for: Education in early life markedly reduces the probability of cognitive impairment in later life in Colombia
Source: Sci Rep. 2020 Oct 19;10:17685. doi: 10.1038/s41598-020-74822-2 (PMC7572407; doi:10.1038/s41598-020-74822-2)
Supplement: Supplementary file 1 — Supplementary information [file 41598_2020_74822_MOESM1_ESM.docx]

**Education in early life markedly reduces the probability of cognitive impairment in later life in Colombia**

Gary O’Donovan,^1^* Mark Hamer,^2^ Olga L Sarmiento,^1^ and Philipp Hessel^3^

*^1^Facultad de Medicina, Universidad de los Andes, Bogotá, Colombia; ^2^Institute of Sport, Exercise and Health, University College London, London, UK; ^3^Escuela de Gobierno Alberto Lleras Camargo, Universidad de los Andes, Bogotá, Colombia.*

*Correspondence: Dr Gary O’Donovan, Facultad de Medicina, Universidad de los Andes, Carrera 1, 18A-12, Bogotá 111711, Colombia. Email: g.odonovan@uniandes.edu.co.

**Table S1. Participants’ characteristics in SABE and in the sub-samples**

| Variable | SABE (n) | Main analysis (n) | Sensitivity analysis (n) |
| --- | --- | --- | --- |
| Shorter MMSE score (mean±SD) | 14.9±3.9 (23,694) | 16.6±1.9 (16,505)* | 15.4±3.5 (20,174)* |
| Age, years (mean±SD) | 70.8±8.2 (23,694) | 68.9±6.9 (16,505)* | 70.0±7.7 (20,174)* |
| Male, % | 42.68 (23,694) | 45.27 (16,505)* | 44.18 (20,174)* |
| Height (mean±SD) | 156.42±8.89 (20,585) | 156.93±8.83 (16,505)* | 156.44±8.89 (20,174) |
| Some primary education, % | 57.04 (23,601) | 59.02 (16,505)* | 57.47 (20,174) |
| Years of education (mean±SD) | 3.59±3.82 (23,500) | 4.07±4.00 (16,452)* | 3.68±3.85 (20,098)† |
| “Normal” economic situation of family during childhood, % | 42.32 (18,904) | 42.42 (16,505) | - |
| “Good” self-rated health during childhood, % | 89.62 (18,964) | 89.70 (16,505) | - |
| Current income less than minimum wage, % | 57.71 (23,339) | 55.64 (16,505)* | 57.60 (20,174) |
| Married or with partner, % | 53.02 (23,684) | 57.60 (16,505)* | 54.93 (20,174)* |
| Current or ex-smoker, % | 51.87 (23,686) | 52.61 (16,505) | 52.60 (20,174)† |
| Alcohol drinker, % | 11.81 (23,677) | 14.18 (16,505)* | 12.74 (20,174)* |
| “Good” self-rated nutritional status, % | 72.31 (18,629) | 72.28 (16,505) | - |
| Physically active, % | 52.23 (23,669) | 59.66 (16,505)* | 55.64 (20,174)* |

*Significantly different to entire sample, p<0.001. †Significantly different to entire sample, p<0.05. Cognitive impairment was defined as the lowest tertile of the shorter version of the mini-mental state examination (MMSE) in the main analysis and as a score of 12 or less from 19 in the sensitivity analysis. Economic situation of family during childhood, self-rated health during childhood, and self-rated nutritional status could not be included in the sensitivity analysis because the proxy interviewee was not asked about these variables.

**Table S2. Participants’ characteristics in the main analysis according to education level**

| Variable | Education (n) |  |  |
| --- | --- | --- | --- |
|  | None (2,606) | Some primary (9,742) | Some secondary or more (4,157) |
| Shorter MMSE score (mean±SD) | 15.76±1.87 | 16.52±±1.91* | 17.35±1.73*† |
| Age, years (mean±SD) | 70.60±7.36 | 69.22±6.93* | 67.12±6.28*† |
| Male, % | 48.81 | 44.12* | 45.75*‡ |
| Height (mean±SD) | 156.32±8.83 | 156.20±8.71 | 159.00±8.77*† |
| “Normal” economic situation of family during childhood, % | 37.22 | 43.41* | 43.35* |
| “Good” self-rated health during childhood, % | 89.22 | 89.29 | 90.96*† |
| Current income less than minimum wage, % | 73.41 | 60.63* | 32.79*† |
| Married or with partner, % | 55.10 | 58.01* | 58.22* |
| Current or ex-smoker, % | 62.93 | 52.27* | 46.96*† |
| Alcohol drinker, % | 10.32 | 13.27* | 18.74*† |
| “Good” self-rated nutritional status, % | 67.54 | 69.92* | 80.78*† |
| Physically active, % | 55.30 | 57.75* | 66.88*† |

*Significantly different to no education, p<0.001. †Significantly different to some primary education, p<0.001. ‡Significantly different to some primary education, p<0.05. Cognitive impairment was defined as the lowest tertile of the shorter version of the mini-mental state examination (MMSE) in the main analysis.

**Table S3. Participants’ characteristics in the sensitivity analysis according to education level**

| Variable | Education (n) |  |  |
| --- | --- | --- | --- |
|  | None (4,218) | Some primary (11,593) | Some secondary or more (4,363) |
| Shorter MMSE score (mean±SD) | 13.11±4.19 | 15.52±3.23* | 17.08±2.27*† |
| Age, years (mean±SD) | 72.43±8.19 | 70.14±7.56* | 67.44±6.63*† |
| Male, % | 44.95 | 43.36* | 45.61‡ |
| Height (mean±SD) | 155.37±8.87 | 155.91±8.76§ | 158.90±8.82*† |
| Current income less than minimum wage, % | 73.02 | 61.24* | 33.00*† |
| Married or with partner, % | 50.01 | 55.85* | 57.92*† |
| Current or ex-smoker, % | 61.24 | 51.76* | 46.46*† |
| Alcohol drinker, % | 8.68 | 12.12* | 18.31*† |
| Physically active, % | 48.46 | 54.43* | 65.80*† |

*Significantly different to no education, p<0.001. †Significantly different to some primary education, p<0.001. ‡Significantly different to some primary education, p<0.01. §Significantly different to no education, p<0.01. Cognitive impairment was defined as a mini-mental state examination (MMSE) score of 12 or less from 19 in the sensitivity analysis.
